# Chronic Kidney Disease and the Risk of New-Onset Atrial Fibrillation: A Meta-Analysis of Prospective Cohort Studies

**DOI:** 10.1371/journal.pone.0155581

**Published:** 2016-05-13

**Authors:** Weifeng Shang, Lixi Li, Shuai Huang, Rui Zeng, Liu Huang, Shuwang Ge, Gang Xu

**Affiliations:** 1 Department of Nephrology, Tongji Hospital affiliated with Tongji Medical College, Huazhong University of Science and Technology, Wuhan, China; 2 Department of Oncology, Tongji Hospital affiliated with Tongji Medical College, Huazhong University of Science and Technology, Wuhan, China; Thomas Jefferson University, UNITED STATES

## Abstract

**Objective:**

Recent epidemiological evidence indicates an association between chronic kidney disease (CKD) and the risk of new-onset atrial fibrillation (AF), but the results are inconclusive. This meta-analysis examined the association between CKD and new-onset AF.

**Methods:**

PubMed, EMBASE, the Cochrane Collaboration and the reference lists of relevant articles were searched to identify eligible studies. The random effect model was used to calculate the overall multivariable-adjusted hazard ratio (HR) with its corresponding 95% confidence interval (CI). Associations were tested in subgroups of study characteristics and study quality criteria. We also performed sensitivity analyses and assessments of publishing bias.

**Results:**

Seven prospective cohort studies (n = 400,189 participants) were included in this meta-analysis. Pooled results suggested that CKD was associated with an increased adjusted risk estimate for new-onset AF (HR, 1.47; 95% CI, 1.21–1.78), with significant heterogeneity between these studies (I^2^ = 79.7%, P<0.001). Results were not different in any subgroup except sample size. Stratified analyses found that the diagnostic method of CKD and eGFR (estimated glomerular filtration rate), the number of confounders adjusted for, and study quality explained little of the variation between studies. Sensitivity analysis further demonstrated the results to be robust.

**Conclusions:**

CKD is associated with an increased risk of incident AF. Further research is needed to investigate the biological association between CKD and AF and identify a preventive strategy to decrease the incidence of AF in CKD patients.

## Introduction

Atrial fibrillation (AF) is the most common sustained arrhythmia in the general population, and it carries a threefold greater incidence of heart failure, a fivefold greater risk of stroke, and an increased mortality[1]. Chronic kidney disease (CKD) is defined as the presence of kidney dysfunction or damage, and it affects 10–20% of adults in many places worldwide[2]. CKD is a powerful predictor of cardiovascular prognosis, and decreased estimated glomerular filtration rate (eGFR) is related to an increased risk of various types of cardiovascular disease, such as coronary heart disease, stroke, peripheral artery disease, heart failure, sudden cardiac death, and AF[3, 4]. AF and CKD are major health problems worldwide, and the burden of AF is even bigger in patients with concomitant kidney disease. Numerous studies examined the association between CKD and AF. A meta-analysis of 25 studies demonstrated that the incidence and prevalence of AF in end-stage renal disease (ESRD) patients on dialysis were higher than the general population and associated with an increased risk of stroke and mortality[5]. CKD is an early stage of ESRD, which is more common in general population, and whether the incidence of new-onset AF is higher in CKD patients is not well established. Several prospective studies demonstrated an increased incidence of new-onset AF in patients with CKD[6–11], with an eGFR of <60 ml/min/1.73 m^2^, but not all studies demonstrated a similar association. One study demonstrated that CKD was not associated with new-onset AF in 4,663 participants[12]. Therefore, we conducted the present meta-analysis to qualify and quantify the risk of new-onset AF, which may provide additional intervention methods in this area.

## Methods

This study was conducted and reported according to the Preferred Reporting Items for Systematic Reviews and Meta-Analyses (PRISMA) statement checklist (S1 PRISMA Checklist)[13].

### Data Sources and Searches

PubMed, EMBASE and the Cochrane Central Register of Controlled Trials databases were searched for cohort studies to September 17, 2015, using the terms “chronic kidney disease” or “chronic kidney failure” or “chronic kidney insufficiency” or “chronic kidney dysfunction” or “chronic renal failure” or “chronic renal insufficiency” or “chronic renal dysfunction” or “end-stage kidney disease” or “end-stage renal disease” and “atrial fibrillation” or “atrial flutter” and “risk” or “incidence” or “epidemiology”. Two authors (WS and SG) filtered all eligible papers using these terms and hand-searched the references of the retrieved articles for additional relevant studies. There were no language restrictions. Discrepancies between the two authors were solved by discussing with a third author. We merged retrieved citations using EndNote.

### Inclusion Criteria

Studies were included if they met the following criteria: (1) prospective cohort study involving participants 18 years or older; (2) assessed the incidence of new-onset AF in CKD patients; and (3) provided the multivariable-adjusted hazard ratios (HRs) with the corresponding 95% confidence intervals (CIs) for events associated with CKD versus reference or sufficient information to calculate these values.

### Exclusion Criteria

The following studies were excluded: reviews, editorials, case reports, conference publications, cross-sectional studies, and case-control studies. We selected the latest article or the largest sample size if a cohort study was reported in more than one publication.

### Data Extraction and Quality Evaluation

The following data were extracted: first author’s name, year of publication, study design, country of origin, population source, sample size, women (%), mean age, time period of the study conducted, mean follow-up period, diagnostic method of CKD and AF, eGFR, multivariable-adjusted risk estimates and their 95% CIs, and adjustment factors. We contacted the original author for clarification when needed. Two authors (WS and SG) independently evaluated the quality of each study using the Newcastle-Ottawa Scale (NOS)[14]. The NOS, including selection, comparability and outcome, is a scale for the evaluation of the quality of published non-randomized studies. Articles scoring 0–3, 4–6, and 7–9 were defined as poor, fair and good quality, respectively. Conflicting results were reconciled by discussion.

### Data Synthesis and Analysis

We collected multivariable-adjusted HR and 95% CI to pool the data and evaluated the heterogeneity of the mean difference using the Chi-squared-based Q-statistic test. The random effects model was used to calculate the pooled HRs if the P value of the heterogeneity Q-statistic was less than 0.10[15]. The fixed effects model was selected in all other circumstances[16]. We also quantified the effect of heterogeneity using I^2^ statistics[17]. I^2^ values of 25%, 50% and 75% represented low, moderate and high heterogeneity, respectively. Sources of heterogeneity were explored using subgroup analyses and univariable random effects meta-regression. Stratified analyses were conducted based on study design (community-based or population-based), region (Asian or non-Asian), sample size (<10000 or ≥10000), participant’s average age (<60 or ≥60 years), follow-up time (<7.5 or ≥7.5 years), diagnostic method of CKD and eGFR (yes or no), the number of confounders adjusted for (<11 or ≥11), and study quality (fair or good). Sensitivity analyses were conducted to assess the robustness of results using sequential omission of individual studies[18]. Publication bias was evaluated using Egger’s test[19]. A P value <0.10 was considered significant. We used Stata 10.0 (College Station, TX, USA) for all analyses.

## Results

### Study Selection, Characteristics, and Quality

**Fig 1:**
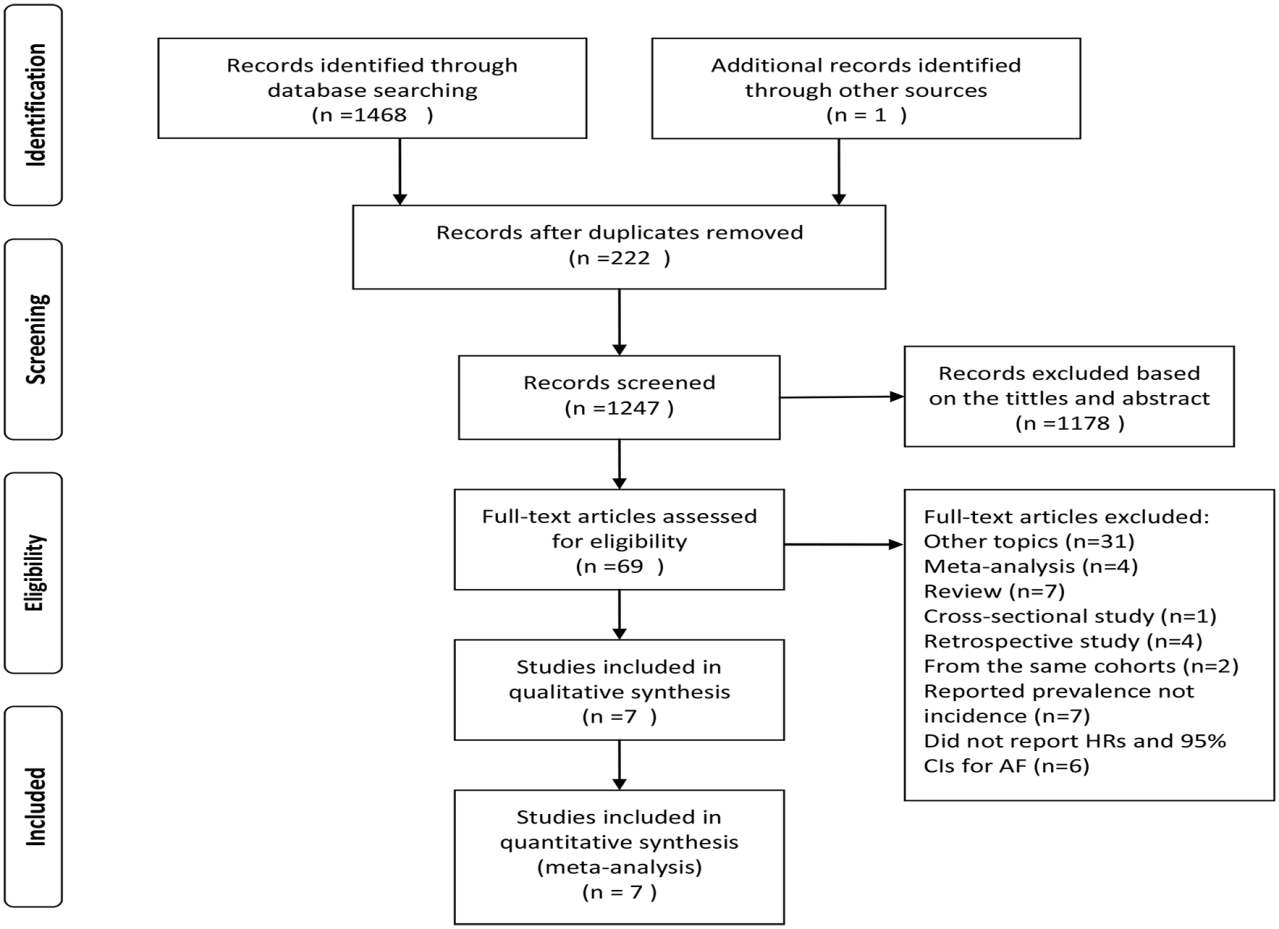
Flow chart of study selection.

Fig 1 shows that our literature search returned 1,469 results for relevant articles, and the full text review retrieved 69 articles, from which we identified 7 prospective cohort studies in the meta-analysis. Table 1 presents the main characteristics of the included studies. Included studies were published in 2009–2015. These articles included 4 population-based studies, 2 community-based studies, and 1 hospital-based study. Three of these studies were conducted in the United States, two studies were performed in Japan, and one study was conducted in Italy. One study enrolled only women, and the remaining studies enrolled both sexes. The average follow-up time ranged from 3.4 to 15.4 years. Definition of CKD only was based on an eGFR<60 ml/min/1.73 m^2^ in 4 studies. The eGFR was calculated using the 4-variable Modification of Diet in Renal Disease (MDRD) Study equation in 5 studies. HRs were obtained from Cox regression model in all studies except Deo et al’s study. All studies adjusted for at least 5 factors for potential confounders. The primary analysis included data for 400,189 participants derived from 7 prospective cohort studies that reported an association between CKD and the incidence of AF. NOS revealed that 5 studies were good quality, and 2 studies were fair quality (S1 Table).

**Table 1:** Study Characteristics.

Study	Watanabe et al. 2009[10]	Deo et al.2010[12]	Horio et al. 2010[7]	Alonso et al. 2011[6]	Sandhu el al. 2012[8]	Sciacqua et al. 2014[9]	Xu et al. 2015[11]
Design	community-based prospective study	population-based prospective study	hospital-based prospective study	population-based prospective study	population-based prospective study	population-based prospective study	community-based prospective study
Country	Japan	America	Japan	United States	United States	Italy	Japan
Population	residents ≥20 years of age	persons≥65 years of age	hypertensive patients	The Atherosclerosis Risk in Communities Study	female health professionals in 1993 who were aged 45 years	Caucasian outpatients	the Ibaraki Prefectural Health Study
Sample Size	223,877	4321	1118	10,328	24,746	3549	132,250
Women (%)	68%	59%	48%	57%	100%	48%	68.5%
Average Age (y)	60.9	75.1	63	62.7	54.6	60.7	59.3
Study Period	1996–2005	1992–2001	1997–2003	1996–2007	1993–2010	1998–2011	1994–2008
Mean Follow-up (y)	5.9	7.4	4.5	Median 10.1	Median 15.4	3.4	13.8
CKD Diagnosis	A baseline normal value and a decline by≥10 ml/min/1.73 m^2^, above the normal age-related, de crease over 10 years, to<60 ml/min/1.73 m^2^; proteinuria (≥ 1+)	eGFR<60 ml/min/1.73 m^2^	eGFR<60 ml/min/1.73 m^2^ and/or the presence of proteinuria (≥ 1+)	National Kidney Foundation guidelines	eGFR<60 ml/min/1.73 m^2^	eGFR<60 ml/min/1.73 m^2^	eGFR<60 ml/min/1.73 m^2^
eGFR	MDRD	MDRD	MDRD	CKD Epidemiology Collaboration equation for cystatin	MDRD	CKD Epidemiology Collaboration	MDRD
AF Diagnosis	ECG	self-report, annual ECG, or by hospital discharge diagnosis	ECG	Hospital discharge codes and death certificates	medical chart review	ECG, hospital discharge diagnoses, and the all-clinical documentation	ECG
Multivariable HR (95%CI)	1.38 (1.14–1.66)	0.92 (0.72–1.19)	2.18 (1.12–3.90)	1.43 (1.19–1.71)	1.37 (1.04–1.82)	1.53 (1.26–1.85)	2.07 (1.73–2.49)
Adjusted Confounders	age, sex, BMI, SBP, DBP, treated hypertension and diabetes	age, gender, race, diabetes, CRP, LDL, HDL, prevalent CHD, prevalent CHF, left ventricular hypertrophy, SBP, DBP, use of ACEI, β-blocker, CCB, and diuretic	age, smoking, use of diuretic, LA diameter, and LV mass index	age, sex, study site, education, height, hsCRP, BMI, SBP, prevalent cardiovascular disease, smoking, alcohol intake, and use of antihypertensive medication	age, SBP, hsCRP, BMI, exercise, assigned treatment, hypertension medication, smoking, alcohol consumption, cholesterol, postmenopausal hormone use, and diabetes	age, gender, smoking, BMI, diabetes, hypertension, hypercholesterole-mia, LVMI, and LAVI	age, sex, SBP, DBP, BMI, total cholesterol, triglyceride, HDL, smoking, alcohol drinking, and diabetes

Abbreviations: CKD, chronic kidney disease; AF, atrial fibrillation; HR, hazard ratio; CI, confidence interval; eGFR, estimated glomerular filtration rate; MDRD, Modification of Diet in Renal Disease; ECG, electrocardiogram; BMI, body mass index; SBP, systolic blood pressure; DBP, diastolic blood pressure; hsCRP, high-sensitivity C-reactive protein; LDL, low-density lipoprotein; HDL, high-density lipoprotein cholesterol; CHF, congestive heart failure; ACEI, angiotensin converting enzyme inhibitor; CCB, calcium channel blocker; LA, left atrial; LV, left ventricular; LVMI, left ventricular mass index; LAVI, left atrial volume index.

### Risk of CKD on AF Events

**Fig 2:**
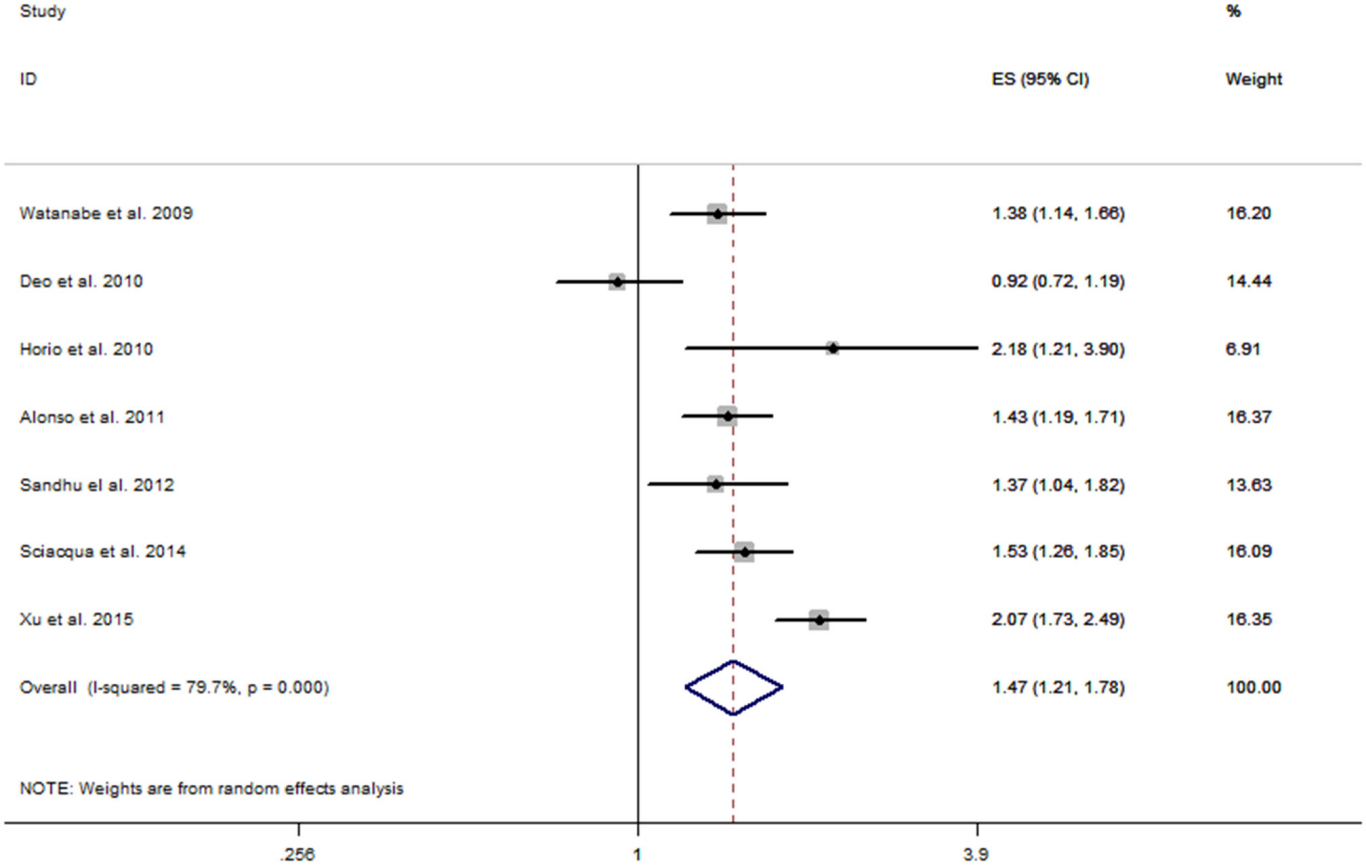
Forest plot of comparisons: CKD (eGFR <60 ml/min/1.73 m^2^) versus eGFR ≥60 ml/min/1.73 m^2^, outcome: AF. Abbreviations: CKD, chronic kidney disease; AF, atrial fibrillation; HR, hazard ratio; CI, confidence interval; eGFR, estimated glomerular filtration rate.

The multivariable-adjusted HR of AF within the 7 individual study populations ranged between 0.92 and 2.18, with an overall multivariable-adjusted HR of 1.47 (95% CI, 1.21–1.78). Significant heterogeneity was observed (I^2^ = 79.7%, P <0.001). Fig 2 shows a forest plot of the multivariable-adjusted HR.

### Subgroup Analyses

No significant difference in the multivariable-adjusted HR was observed between subgroups stratified according to most of the study-level characteristics and study quality, except sample size. Stratification based on sample size indicated that the pooled risks for AF remained significant in studies with a sample size ≥10000 participants. However, no association was identified in studies with a sample size <10000 participants, and the difference was not statistically significant (P = 0.573). Low-to-high heterogeneity remained present in stratified analyses unless eGFR was not calculated using the MDRD Study equation (I^2^ = 0%). We used meta-regression to explore the sources of heterogeneity and found that the diagnostic method of CKD and eGFR, the number of confounders adjusted for, and study quality may be potential sources of heterogeneity (Table 2).

**Table 2:** Subgroup Analyses of AF in CKD.

Subgroup	No. of studies	HR (95% CI)	I^2^ (%)	P value for interaction
Study design				
community-based	2	1.69 (1.14–2.52)	89.2	0.273
population-based	4	1.30 (1.05–1.61)	72.6	
Region				
Asian	3	1.77 (1.27–2.47)	79.8	0.167
Non-Asian	4	1.30 (1.05–1.61)	72.6	
Sample size				
<10000	3	1.38 (0.89–2.14)	84.6	0.573
≥10000	4	1.55 (1.26–1.91)	76.2	
Participant’s average age (years)				
<60	2	1.71 (1.14–2.56)	83	0.360
≥60	5	1.36 (1.12–1.65)	70.5	
Follow-up time, years				
<7.5	4	1.36 (1.04–1.77)	77.3	0.460
≥7.5	3	1.61 (1.23–2.11)	80.1	
Definition of CKD only based on eGFR<60 ml/min/1.73 m^2^				
Yes	4	1.42 (1.02–1.98)	88.9	0.772
No	3	1.44 (1.26–1.64)	6.1	
eGFR was calculated using the MDRD Study equation				
Yes	5	1.47 (1.08–2.00)	86.3	0.972
No	2	1.48 (1.29–1.68)	0	
Adjustment for confounding factors				
<11	3	1.49 (1.28–1.73)	14.2	0.637
≥11	4	1.40 (1.01–1.94)	89	
Study quality				
fair	2	1.60 (1.04–2.47)	49.2	0.666
good	5	1.43 (1.14–1.80)	85.5	

Abbreviations: CKD, chronic kidney disease; AF, atrial fibrillation; HR, hazard ratio; CI, confidence interval; eGFR, estimated glomerular filtration rate; MDRD, Modification of Diet in Renal Disease.

### Sensitivity Analyses and Reporting Bias

Sensitivity analysis indicated that the omission of any of the studies changed estimates between 1.36 (95% CI: 1.16–1.60) and 1.58 (95% CI: 1.35–1.84) (S2 Table). The changes were not significant. However, deletion of the Deo et al.[12] or Xu et al.[11] study reduced the heterogeneity from high to moderate levels. The P value for Egger’s test was 0.855, which suggests a low probability of publication bias.

## Discussion

This study is the first meta-analysis to present new-onset AF risk in patients with CKD. We demonstrated that CKD was associated with an increased risk of new-onset AF with an HR of 1.47 (95% CI, 1.21–1.78). The results of our meta-analysis also revealed a stronger association between CKD and AF in subgroups that were community-based, Asian, sample size (≥10000), participant’s average age (<60 years), and follow-up time (≥7.5 years), but the difference was not statistically significant between subgroups. Notably, there was no significant association between CKD and AF in studies with a sample size <10000 participants.

There was high heterogeneity in the meta-analysis. Subgroup analyses by diagnostic method of CKD revealed that heterogeneity was almost removed in the subgroup where CKD was not defined only based on eGFR<60 ml/min/1.73 m^2^, which may be a potential source of heterogeneity. Heterogeneity was not present in the subgroups where eGFR was not calculated using the MDRD Study equation, which reflected that the diagnostic method of eGFR may also be a main source of heterogeneity. Grouping studies by the number of confounders adjusted for largely reduced heterogeneity in the subgroup (less than 11 factors adjusted for), which suggests that the adjusted confounding factors partially explained the heterogeneity. Stratification by study quality demonstrated that heterogeneity was reduced in studies with fair quality, likely because of the introduction of more complex confounding factors into studies with good quality. These results revealed that diagnostic method of CKD and eGFR, the number of confounders adjusted for, and study quality may constitute a source of heterogeneity. The exclusion of Deo et al.’s or Xu t al.’s study obviously decreased the heterogeneity. These two studies also played a partial role in the heterogeneity.

The relationship between CKD and new-onset AF is not clear, but several potential reasons may explain the observed associations. First, the raising activation of the renin-angiotensin-aldosterone system, which is related to the being of CKD, could cause cause atrial system lesions, including elevated left atrial pressure, atrial dilatation and atrial fibrosis, and further invoke the emergence of AF[4]. Second, it has been demonstrated that there are increasing level of inflammatory markers such as C-reactive protein and fibrinogen in patients with CKD[20–21] and they are associated with the initiation and perpetuation of AF[22–23]. Third, oxidative stress and endothelial dysfunction caused by renal impairment may be involved in the increased risk of new-onset AF in patients with CKD[24]. Forth, CKD may trigger AF via an increased risk of cardiovascular disease[25–27] or activation of the sympathetic nervous system[28]. Additionally, some studies have found AF is also associated with an increased risk of CKD, the two conditions may have a bidirectional association.

Several limitations of this meta-analysis should be noted. First, eGFR was not directly measured, but it was estimated using different eGFR equations, which may lead to misclassification of renal function. Second, AF was diagnosed based on annual ECG recordings in most included studies, which may underestimate the true incidence of AF. Third, our study population included more women than men. Male gender is a risk factor for AF, but most of the included studies were adjusted for gender[29]. Fourth, the type of AF could not be fully discriminated in this analysis. Therefore, we could not evaluate the association between CKD and different AF risk. Fifth, the results of our meta-analysis are not based on individual patient data, but all of included studies adjusted adequately for potential confounders, which reduces the possibility that other risk factors affected the association between CKD and AF. Finally, a cause-and-effect could not be established because the results of the study were based on cohort studies, and residual confounding factors may be present.

In conclusion, our meta-analysis demonstrates CKD is associated with an increased multivariable-adjusted HR for new-onset AF. Our results increase the awareness of incidence of AF in CKD patients among the public, policy makers and health care professionals. Further efforts should be made to explore the potential biological mechanism and identify a preventive strategy to decrease the incidence of AF in CKD patients. Large-scale and long-term randomized controlled trials in various populations are further warranted to reveal the strength of this association.

## Supporting Information

S1 MOOSE Checklist MOOSE Checklist. (DOC)

S1 PRISMA Checklist PRISMA Checklist. (DOC)

S1 Table Assessment of study quality. (DOC)

S2 Table Sensitivity analysis. (DOC)
