# Supplementary material for: Chronic Kidney Disease and the Risk of New-Onset Atrial Fibrillation: A Meta-Analysis of Prospective Cohort Studies
Source: PLoS One. 2016 May 13;11(5):e0155581. doi: 10.1371/journal.pone.0155581 (PMC4866731; doi:10.1371/journal.pone.0155581)
Supplement: S1 MOOSE Checklist — (DOC) [file pone.0155581.s001.doc]

**Meta-analysis of Observational Studies in Epidemiology (MOOSE) Checklist**

**Chronic kidney disease and the risk of new-onset atrial fibrillation: a meta-analysis of prospective cohort studies**

| **Criteria** | | **Brief description of how the criteria were handled in the meta-analysis** |
| --- | --- | --- |
| **Reporting of background should include** | |  |
| √ | Problem definition | CKD and AF are major health problems worldwide.  Several prospective studies have showed increased incidence of new-onset AF in patients with CKD, with a eGFR of <60 mL/min/1.73 m2, but not all studies have shown a similar association. Given these inconsistent results, we conducted the present meta-analysis. |
| √ | Hypothesis statement | CKD increases the risk of new-onset AF. |
| √ | Description of study outcomes | new-onset AF |
| √ | Type of exposure or intervention used | CKD |
| √ | Type of study designs used | Prospective cohort study studies that estimating the incidence of new-onset AF among CKD patients with the multivariable-adjusted hazard ratios with the corresponding 95% confidence intervals. |
| √ | Study population | 18 years or older |
| **Reporting of search strategy should include** | |  |
| √ | Qualifications of searchers | The two experienced investigators (SG and WS ) are indicated in the authors list. |
| √ | Search strategy, including time period included in the synthesis and keywords | PubMed,EMBASE and the Cochrane Central Register of Controlled Trials databases were searched for all articles published before September 17, 2015. Keywords: “CKD and AF” |
| √ | Databases and registries searched | PubMed, EMBASE and the Cochrane Central Register of Controlled Trials |
| √ | Search software used, name and version, including special features | We did not employ a search software. Endnote was used  to merge retrieved citations. |
| √ | Use of hand searching | We hand-searched references of retrieved papers for  additional references. |
| √ | List of citations located and those excluded, including justifications | Figure 1 |
| √ | Method of addressing articles published in languages other than English | We placed no restrictions on language; We were able to obtained all articles potentially eligible for inclusion in English language |
| √ | Method of handling abstracts and unpublished studies | We did not include unpublished or abstract only  publications |
| √ | Description of any contact with authors | When needed, we contacted the original anthor for clarification. |
| **Reporting of methods should include** | |  |
| √ | Description of relevance or appropriateness of studies assembled for assessing the hypothesis to be tested | Detailed inclusion and exclusion criteria are described in the paper. |
| √ | Rationale for the selection and coding of data | The following data were extracted : first author’s name, year of publication, study design, country of origin, population source, sample size, women (%), mean age, time period of the study conducted, mean follow-up period, method of CKD and AF, eGFR, multivariable-adjusted risk estimates and their 95% CIs, and adjustment factors. |
| √ | Assessment of confounding | We conducted subgroup analyses and univariable random-effects meta-regression. |
| √ | Assessment of study quality, including blinding of quality assessors; stratification or regression on possible predictors of study results | We used a modified version of the Newcastle Ottawa Scale (NOS) to assess the quality of each study. |
| √ | Assessment of heterogeneity | We used the P and I^2^ value to assess heterogeneity |
| √ | Description of statistical methods in sufficient detail to be replicated | We mentioned type of analysis we used (meta-analysis ,subgroup meta-analysis and meta-regression ) and type of software we used Stata 10.0 (College Station, TX, USA) |
| √ | Provision of appropriate tables and graphics | Table1 showing study characteristics, Table 2 showing results of subgroup analysis, Table S1 showing results of quality assessment, Table S2 showing the results of sensitivity analysis, Figure 1 showing literature search flow diagram, Figure 2 showing forest plot |
| **Reporting of results should include** | |  |
| √ | Graph summarizing individual study estimates and overall estimate | Figure 2 |
| √ | Table giving descriptive information for each study included | Table 1 |
| √ | Results of sensitivity testing | Table S2 |
| √ | Indication of statistical uncertainty of findings | HR, 95% CI, I^2^ and P |
| **Reporting of discussion should include** | |  |
| √ | Quantitative assessment of bias | Results of Funnel plot and subgroup analyses were discussed |
| √ | Justification for exclusion | Studies were excluded: reviews, editorials, case reports, conference publications, cross-sectional studies, and case-control studies. We selected the latest article or the largest sample size if a cohort study was reported in more than one publication. |
| √ | Assessment of quality of included studies | Table S1 |
| **Reporting of conclusions should include** | |  |
| √ | Consideration of alternative explanations for observed results | First, eGFR was not directly measured, but it was estimated using different eGFR equations, which may lead to misclassification of renal function. Second, AF was diagnosed based on annual ECG recordings in most included studies, which may underestimate the true incidence of AF. Third, our study population included more women than men. Male gender is a risk factor for AF, but most of the included studies were adjusted for gender. Fourth, the type of AF could not be fully discriminated in this analysis. Therefore, we could not evaluate the association between CKD and different AF risk. Fifth, the results of our meta-analysis are not based on individual patient data, but all of included studies adjusted adequately for potential confounders, which reduces the possibility that other risk factors affected the association between CKD and AF. Finally, a cause-and-effect could not be established because the results of the study were based on cohort studies, and residual confounding factors may be present. |
| √ | Generalization of the conclusions | our meta-analysis demonstrates CKD is associated with an increased multivariable-adjusted HR for new-onset AF. |
| √ | Guidelines for future research | Further efforts should be made to explore the potential biological mechanism and search for the preventive strategy to decrease the risk of AF in CKD patients. Large-scale and long-term randomized controlled trials in various populations are further warranted to show the strength of this association. |
| √ | Disclosure of funding source | This work was supported by the National Nature Science Foundation of China (NSFC) (No. 81200531 for Shuwang Ge; No. 81470948 and 81270770 for Gang Xu). |
