# Supplementary material for: Chronic Kidney Disease and the Risk of New-Onset Atrial Fibrillation: A Meta-Analysis of Prospective Cohort Studies
Source: PLoS One. 2016 May 13;11(5):e0155581. doi: 10.1371/journal.pone.0155581 (PMC4866731; doi:10.1371/journal.pone.0155581)
Supplement: S1 Table — (DOC) [file pone.0155581.s003.doc]

**Table S1. Assessment of study quality.**

| **References** | **Quality indications form of Newcastle-Ottawa Scale** | | | | | | | | | **Total stars** |
| --- | --- | --- | --- | --- | --- | --- | --- | --- | --- | --- |
|  | **1** | **2** | **3** | **4** | **5A** | **5B** | **6** | **7** | **8** |  |
| Watanabe et al. 2009 | Yes | Yes | Yes | Yes | Yes | Yes | Yes | No | Yes | 8 |
| Deo et al.2010 | Yes | Yes | Yes | Yes | Yes | Yes | Yes | No | No | 7 |
| Horio et al. 2010 | Yes | Yes | Yes | No | No | Yes | Yes | No | No | 5 |
| Alonso et al. 2011 | Yes | Yes | Yes | Yes | Yes | Yes | Yes | Yes | No | 8 |
| Sandhu el al. 2012 | No | Yes | Yes | Yes | No | Yes | Yes | Yes | No | 6 |
| Sciacqua et al. 2014 | Yes | Yes | Yes | Yes | Yes | Yes | Yes | No | No | 7 |
| Xu et al. 2015 | Yes | Yes | Yes | Yes | Yes | Yes | Yes | Yes | No | 8 |

For cohort studies: 1, exposed cohort truly or somewhat representative; 2, nonexposed cohort drawn from the same community as the exposed cohort; 3, ascertainment of exposure; 4, outcome of interest not present at start; 5A, study controls for age and sex; 5B, study controls for ≥3 additional risk factors; 6, assessment of outcome (independent blind assessment or record linkage); 7, follow-up ≥7.5 y; 8, complete accounting for cohorts or subjects lost to follow-up unlikely to introduce bias
