# Supplementary material for: Chronic Kidney Disease and the Risk of New-Onset Atrial Fibrillation: A Meta-Analysis of Prospective Cohort Studies
Source: PLoS One. 2016 May 13;11(5):e0155581. doi: 10.1371/journal.pone.0155581 (PMC4866731; doi:10.1371/journal.pone.0155581)
Supplement: S2 Table — (DOC) [file pone.0155581.s004.doc]

**Table S2. Sensitivity analysis.**

| Study omitted | HR | 95%CI | | I^2^ (%) | P |
| --- | --- | --- | --- | --- | --- |
| Watanabe et al. 2009 | 1.49 | 1.18 | 1.88 | 82.6 | <0.001 |
| Deo et al.2010 | 1.58 | 1.35 | 1.84 | 64 | 0.016 |
| Horio et al. 2010 | 1.42 | 1.17 | 1.74 | 82.1 | <0.001 |
| Alonso et al. 2011 | 1.48 | 1.16 | 1.87 | 83 | <0.001 |
| Sandhu el al. 2012 | 1.48 | 1.19 | 1.85 | 82.9 | <0.001 |
| Sciacqua et al. 2014 | 1.46 | 1.15 | 1.84 | 83 | <0.001 |
| Xu et al. 2015 | 1.36 | 1.16 | 1.60 | 63.1 | 0.019 |

Abbreviations: HR, hazard ratio; CI, confidence interval.
